# The beneficial effects of curcumin in cirrhotic rats with portal hypertension

**DOI:** 10.1042/BSR20171015

**Published:** 2017-12-15

**Authors:** Shao-Jung Hsu, Jing-Yi Lee, Te-Yueh Lin, Yu-Hsin Hsieh, Hui-Chun Huang, Fa-Yauh Lee, Han-Chieh Lin, Ming-Chih Hou, Shou-Dong Lee

**Affiliations:** 1 Faculty of Medicine, National Yang-Ming University School of Medicine, Taipei, Taiwan; 2 Institute of Pharmacology, National Yang-Ming University School of Medicine, Taipei, Taiwan; 3 Division of Gastroenterology and Hepatology, Department of Medicine, Taipei Veterans General Hospital, Taipei, Taiwan; 4 Division of General Medicine, Department of Medicine, Taipei Veterans General Hospital, Taipei, Taiwan; 5 Endoscopy Center for Diagnosis and Treatment, Taipei Veterans General Hospital, Taipei, Taiwan; 6 Division of Gastroenterology, Department of Medicine, Cheng Hsin General Hospital, Taipei, Taiwan

**Keywords:** angiogenesis, curcumin, liver cirrhosis, portal hypertension, portosystemic collaterals

## Abstract

In liver cirrhosis with portal hypertension, the uneven distribution of vasoactive substances leads to increased intrahepatic vascular resistance and splanchnic vasodilatation. Angiogenesis also induces increased portal inflow and portosystemic collaterals. The collaterals may induce lethal complications such as gastroesophageal variceal hemorrhage, but the therapeutic effect of vasoconstrictors is still suboptimal due to poor collateral vasoresponsivenss. Curcumin has aroused much attention for its antifibrosis, vasoactive, and anti-angiogenesis actions. However, whether it affects the aforementioned aspects is unknown. Liver cirrhosis was induced by common bile duct ligation (CBDL) in Sprague–Dawley rats. Sham-operated rats were controls. CBDL and sham rats were randomly allocated to receive curcumin (600 mg/kg per day) or vehicle since the 15th day after BDL. On the 29th day, portal hypertension related parameters were surveyed. Portosystemic collateral *in situ* perfusion was performed to evaluate vascular activity. Chronic curcumin treatment decreased portal pressure (PP), cardiac index (CI) and increased systemic vascular resistance (SVR) in cirrhotic rats. In splanchnic system, curcumin decreased superior mesenteric artery (SMA) flow and increased SMA resistance. Mesenteric angiogenesis was attenuated by curcumin. Acute administration of curcumin significantly induced splanchnic vasoconstriction. The mesenteric protein expressions of p-endothelial nitric oxide synthase (eNOS), cyclooxygenase (COX) 2 (COX2), vascular endothelial growth factor (VEGF), p-VEGF receptor 2 (VEGFR2), and p-Erk were down-regulated. In collateral system, curcumin decreased portosystemic shunting and induced vasoconstriction. In conclusion, chronic curcumin administration in cirrhotic rats ameliorated portal hypertension related hemodynamic derangements and portosystemic collaterals. Curcumin also attenuated splanchnic hyperdynamic circulation by inducing vasoconstriction through inhibition of eNOS activation and by decreasing mesenteric angiogenesis via VEGF pathway blockade.

## Background

Liver cirrhosis is an end-stage condition induced by acute or chronic liver injury. The hemodynamic changes characterized by increased intrahepatic resistance and splanchnic vasodilatation with increased portal inflow induce portal hypertension. When the increased portal inflow confronts the difficulty in entering the liver, the portosystemic collaterals develop gradually with an attempt to divert the stagnant blood flow. Amongst them, gastroesophageal varices bleeding could be lethal and the efficacy of pharmacological treatment is still suboptimal. Traditionally, splanchnic vessel dilatation was thought as a key factor. However, recent studies have indicated that angiogenesis participates in the development and maintenance of splanchnic hyperemia and portosystemic collateral vascular bed in portal hypertension [1,2]. Furthermore, angiogenesis blockade could be beneficial in the control of portal hypertension [3,4].

Portal pressure (PP) is mainly determined by the interaction between hepatic and splanchnic systems. During the progression of liver cirrhosis, hepatic fibrosis and regeneration nodules compress intrahepatic vessels and increased hepatic resistance, which is a major pathophysiological factor of portal hypertension. Furthermore, the splanchnic flow increases because of abnormal angiogenesis (structural component) and vascular dilatation (functional component), which elevate portal inflow and subsequently PP. The contractility and amount of collaterals also influence PP and bring lethal complications. To contract collateral vessels and to decrease the amount of shunting are therefore fundamental in the control of portal hypertension related complications. However, the therapeutic efficacy of vasoconstrictors is still limited by their side effects [5] and poorer vasoresponsiveness during acute hemorrhage in portal hypertension, which were mainly ascribed to nitric oxide (NO) [6].

Curcumin (curcumin [1,6-heptadiene3,5-dione,1,7-bis(4-hydroxy-3-methoxyphenyl); C_21_H_20_O_6_]), an active ingredient in the dietary agent turmeric, has potent antiproliferative, anti-inflammatory, and anti-angiogenic properties [7]. It has been consumed daily throughout Asian countries over centuries without significant toxicity [8]. Curcumin administered at the onset of liver disease has been proved to alleviate liver fibrosis in animals [9]. Curcumin also suppressed angiogenesis *in vivo* [10]. Furthermore, curcumin exerts various vasoactive effects through different mechanisms [11]. However, the relevant influence of curcumin on portal hypertension and related derangements have not been surveyed. The present study thus aimed to evaluate the effects of curcumin on the aforementioned aspects in cirrhotic rats.

## Materials and methods

### Animal model: common bile duct ligation

Male Sprague–Dawley rats (260–280 g) were caged at 24°C with a 12-h light/dark circle and allowed free access to food and water. Secondary biliary cirrhosis was induced by common bile duct ligation (CBDL) [12]. In brief, under ketamine anesthesia (100 mg/kg, intramuscularly), the common bile duct was exposed through a midline abdominal incision and doubly ligated with 3-0 silk. The section between the ligatures was cut. The incision was then closed and the animal allowed to recover. A high yield of secondary biliary cirrhosis was noted 4 weeks later [13]. Weekly vitamin K injection (50 μg/kg, intramuscularly) was applied to avoid coagulation defect.

The Taipei Veterans General Hospital Animal Committee approved the study (grant number IACUC 2012-106). All animals received humane care according to the criteria outlined in the ‘Guide for the Care and Use of Laboratory Animals’ prepared by the National Academy of Sciences and published by the National Institutes of Health (NIH Publication 86-23, revised 1985).

### Experiment design

#### Effects of chronic curcumin administration on portal hypertension related parameters

To determine the role of curcumin in the progression of cirrhosis and portal hypertension, CBDL and sham rats were randomly allocated to receive curcumin (600 mg/kg/day, oral gavage) or vehicle (distilled water, DW) for 2 weeks since the 15th day after CBDL or sham operation. On the 29th day after operations, the following parameters were determined: (i) systemic hemodynamic effects of curcumin; (ii) effects of curcumin on hepatic system; (iii) effects of curcumin on splanchnic system: mesenteric vascular density and protein expressions; (iv) effects of curcumin on collateral vascular bed: portosystemic shunting. Part (iv) was performed in parallel groups because of technical requirement.

#### Effects of curcumin on splanchnic system: acute effects of curcumin on splanchnic hemodynamics

To determine the acute effects of curcumin on splanchnic vascular hemodynamics, CBDL rats were applied. On the 29th day after operations, a single dose of curcumin (1000 mg/kg, oral gavage) or vehicle was given. Splanchnic hemodynamics were measured at 5, 25, and 45 min after curcumin administration.

#### Effects of curcumin on collaterals: acute effect of curcumin on vascular contractility of collateral vascular bed

To further explore the effects of curcumin on the vasoresponsiveness to arginine vasopressin (AVP) in collateral vascular bed, *in situ* collateral perfusion was performed with Krebs solution (vehicle) or curcumin (10 mM) preincubation for 20 min. Then the perfusion pressure changes to AVP (10^−10^, 10^−9^, 3 × 10^−9^, 10^−8^, 3 ×10^−8^, and 10^−7^ M) were evaluated.

### Measurement of systemic and portal hemodynamics

The right femoral artery was cannulated with a PE-50 catheter that was connected to a Spectramed DTX transducer (Spectramed Inc., Oxnard, CA, U.S.A.). Continuous recordings of mean arterial pressure (MAP), heart rate (HR), and PP were performed on a multichannel recorder (model RS 3400, Gould Inc., Cupertino, CA, U.S.A.). The external zero reference was placed at the level of the midportion of the rat. The abdomen was then opened with a midline incision, and a mesenteric vein was cannulated with a PE-50 catheter connected to a Spectramed DTX transducer. The abdominal cavity was then closed and the PP was recorded on a Gould model RS 3400 recorder [14,15].

The superior mesenteric artery (SMA) was identified at its aortic origin and a 5-mm segment was gently dissected free from surrounding tissues. An ultrasonic flow transducer was placed around the SMA and secured with a 4-0 silk tie threaded through the cuff, and the flow was detected through a small animal blood flow meter (TS420, Transonic Systems Inc., NY, U.S.A.).

Cardiac output (CO) was measured by thermodilution, as previously described [16]. Briefly, a thermistor was placed in the aortic arch just distal to the aortic valve and the thermal indicator (100 μl of normal saline) was injected into the right atrium through a PE-50 catheter. The aortic thermistor was connected to a Columbus Instruments Cardiotherm 500-AC-R (Columbus Instruments International Co., OH, U.S.A.). Five thermodilution curves were obtained for each CO measurement. The final value was obtained from the arithmetic mean. Cardiac index (CI, ml/min/100 g body weight (BW)) was calculated as CO per 100 g BW: systemic vascular resistance (SVR, mmHg/ml/min/100 g BW) was calculated by dividing MAP by CI. SMA resistance (mmHg/ml/min/100 g BW) was calculated by (MAP-PP)/SMA flow per 100 g BW [16].

### Hepatic fibrosis determination with Sirius Red staining

The liver paraffin section was stained with Sirius Red staining kit (Polysciences Inc., Warrington, PA, U.S.A.). ImageJ (available for downloading from the National Institutes of Health (http://rsb.info.nih.gov/ij/)) was used to measure the percentage of Sirius Red stained area. Briefly, gray-scale image was used, then the red-stained collagen was isolated using thresholding function. After that, the thresholded area was measured and shown as the percentage of thresholded area per image.

### Hematoxylin and Eosin staining

The tissue was fixed in 10% formalin, embedded in paraffin, sectioned in 5 μm, and stained with Hematoxylin and Eosin (H&E).

### Immunofluorescence study for the hepatic and mesenteric vascular density

Hepatic and mesenteric angiogenesis were quantitated by CD31-labeled microvascular networks in rat liver and mesenteric connective tissue windows according to the previous study [3,4]; (×100)-magnification immunofluorescent images were assessed using an upright fluorescent microscope (AX80, Olympus, Japan) and thresholded by ImageJ software. The vascular length and area was manually measured with the pencil tool and histogram function, respectively. The unit of vascular length per unit area of mesenteric window would be μm/μm^2^ = μm^−1^ and the vascular area per unit area of mesenteric window, actually, could be pixel/pixel without being converted into μm^2^/μm^2^.

### Western blotting

Mesentery was immediately frozen in liquid nitrogen and stored at −80°C until required. The protein extracts were made by pulverizing in grinder with liquid nitrogen, then using a ratio of 1 ml of lysis buffer (PBS containing 1% NP-40, 0.5% sodium deoxycholate, 0.1% SDS, and 0.05% protease inhibitor cocktail solution (Roche Diagnostics GmbH, Penzberg, Germany) for each 100-mg sample. Protein concentration was determined for each sample by the Bradford method [17]. An aliquot of 20–40 µg protein from each sample that dissolved in sample buffer (63 mmol/l of Tris/HCl, pH 6.8, containing 2% SDS, 10% glycerol, 5% 2-mercaptoethanol, and 0.005% Bromophenol Blue), and 10 µg positive control were separated on denaturing SDS/polyacrylamide gels (10%) by electrophoresis (Mini-PROTEAN® 3 Cell, Bio–Rad Laboratories, Hercules, CA, U.S.A.). Prestained proteins markers (SDS-PAGE Standards, Bio–Rad Laboratories) were used for molecular weight determinations. Proteins were transferred on to a PVDF membrane (Immum-Blot™ PVDF Membrane, Bio–Rad Laboratories) by a semidry electroblotting system (Trans-Blot® SD Semi-dry Electrophoretic Transfer Cell, Bio–Rad Laboratories) for 1.5 h at 4°C. To block non-specific binding, membranes were blocked for 30 min with 3% non-fat dry milk in TBS-T, pH 7.4 (25 mmol/l Tris base, 137 mmol/l NaCl, 2.7 mmol/l KCl, 1% Tween 20). Blots were incubated with the primary antibody (inducible NO synthase (iNOS) (Millipore, Billerica, MA, U.S.A.) used in 1:1000 dilution from Millipore AB5382; p-Erk used in 1:1000 dilution from Signaling 4370; Erk used in 1:1000 dilution from Millipore 05-1152; vascular endothelial growth factor (VEGF) used in 1:500 dilution from Genetex 59912; VEGF receptor 2 (VEGFR2) used in 1:1000 dilution from Cell Signaling 9698; p-VEGFR2 used in 1:1000 dilution from Origene TA309974; cyclooxygenase (COX) 1 (COX1) used in 1:1000 dilution from Cell Signaling 9896; COX2 used in 1:1000 dilution from Thermo PA5-27283), diluted with 5% non-fat dry milk in TBS-T, then washed. After that, the blots were incubated with the secondary antibody diluted with 5% non-fat dry milk in TBS-T (peroxidase-labeled anti-rabbit IgG, 1:6000, room temperature for 60 min for iNOS, p-Erk, VEGF, VEGFR2, COX-1, and COX-2, Millipore AP132P; peroxidase-labeled anti-mouse IgG, 1:6000, room temperature for 60 min for Erk, Millipore AP124P) and washed. Subsequent detection of the specific proteins was performed by ECL (BCIP/NBT solution, Amresco Co., Ohio, U.S.A.). With a computer-assisted video densitometer and digitalized software (Kodak Digital Science™ ID Image Analysis Software, Eastman Kodak Co., Rochester, NY, U.S.A.), the blots were scanned, photographed, and the signal intensity (integral volume) of the appropriate band was analyzed.

### Color microsphere method for portosystemic shunting degree analysis

Portosystemic shunting degree was determined using the technique described by Chojkier and Groszmann [18] and substituting color for radioactive microspheres; 30000 of 15-μm yellow microspheres (Dye Track; Triton Technology, San Diego, CA, U.S.A.) were slowly injected into the spleen. The rats were killed, and the livers and lungs were dissected and placed into new polypropylene centrifuge tubes. The number of microspheres in each tissue was determined following the protocol provided by the manufacturer. In brief, 3000 blue microspheres (Dye Track; Triton Technology) were added to each tube as an internal control. Tissue was digested overnight with 1 M KOH at 60°C and thoroughly sonicated. After centrifugation, the supernatant was removed, and the pellet was washed once with 10% Triton X-100 and twice with acidified ethanol. At the end of the process, a minimum pellet containing the microspheres was allowed to dry overnight. The color of the microspheres was diluted with 200 μl of acidified Cellosolve acetate (Spectrum Chemicals, Gardens, CA, U.S.A.). The absorbance of the solution was read at 448-nm wavelength (yellow) and 670-nm wavelength (blue) in a spectrophotometer (Shimadzu, Columbia, MD, U.S.A.), and the number of microspheres was calculated by comparison with standards. Spillover between wavelengths was corrected with the matrix inversion technique. Portosystemic shunting was calculated as lung microspheres/(liver microspheres + lung microspheres). Assuming a worst-case scenario in which two-thirds of the microspheres remain trapped in the spleen, this technique detects a minimum shunt of 3.5%. Studies using color microspheres have been shown to provide results similar to those using radioactive microspheres [19].

### Portosystemic collateral system perfusion

The *in situ* perfusion system was performed as previously described [20]. Briefly, both jugular veins were cannulated with 16-gauge Teflon cannulas. The abdomen was then opened and an 18-gauge Teflon cannula was inserted in the distal SMV and fixed with cyanoacrylate glue. To exclude the liver from perfusion, the second loose ligature around the portal vein was tied. All the experiments were performed 20 min after starting perfusion at a constant rate of 12 ml/min. In each individual preparation, after testing experimental agents, the contracting capability of the portosystemic collateral vessels was challenged with 125 mM potassium chloride solution at the end of experiments.

### Drugs

Curcumin was purchased from Sigma Chemical Co. (St. Louis, MO. U.S.A.). All solutions were freshly prepared on the days of experiment.

### Statistical analysis

All results are expressed as mean ± S.E.M. The Shapiro–Wilk normality test was applied to check the distribution pattern of the data. Most of the datasets were normally distributed except for liver and renal biochemistry data. They were analyzed with Mann–Whitney test and the others with *t* test or one-way ANOVA test as appropriate. SPSS 21 for Windows (SPSS Inc., Chicago, IL, U.S.A.) was used. Results are considered statistically significant at a two-tailed *P*-value less than 0.05.

## Results

### Systemic effects of curcumin

#### BW and portal hypertension related hemodynamics

Table 1 shows the systemic and portal hemodynamic parameters of DW-treated rats, either sham- or CBDL operated. In DW-treated rats, cirrhotic rats had significantly lower BW, higher PP, lower SVR and SMA resistance, and higher CI and SMA flow as compared with the sham rats.

**Table 1:** BW and hemodynamic parameters in sham or CBDL rats with DW or curcumin treatment

	Sham DW	Sham curcumin	CBDL DW	CBDL curcumin
	*n*=5	*n*=5	*n*=7	*n*=8
**BW (g)**	425 ± 7	415 ± 8	367 ± 11^†^	390 ± 10
**MAP (mmHg)**	110 ± 4	111 ± 4	98 ± 6	92 ± 6
**HR (beats/min)**	329 ± 9	306 ± 11	294 ±19	256 ± 16
**PP (mmHg)**	6.8 ± 1.0	6.7 ± 1.0	15.4 ± 1.0^†^	11.2 ± 1.2*
**CI (ml/min/100 g)**	22.7 ± 1.2	22.9 ± 0.9	38.0 ± 1.6^†^	26.9 ± 3.1*
**SVR (mmHg/ml/min/100 g)**	5.0 ± 0.4	4.7 ± 0.1	2.5 ± 0.2^†^	3.1 ± 0.2*,^‡^
**SMA flow (ml/min/100 g)**	2.6 ± 0.1	2.5 ± 0.1	5.5 ± 0.3^†^	3.9 ± 0.4*
**SMA resistance (mmHg/ml/min/100 g)**	39.4 ± 2.1	40.7 ± 3.2	15.3 ± 1.5^†^	22.2 ± 2.5*,^‡^

* *P*<0.05, curcumin-treated group compared with parallel DW-treated group;

† *P*<0.05, DW-treated CBDL group compared with DW-treated sham group;

‡ *P*<0.05, curcumin-treated CBDL group compared with curcumin-treated sham group.

CBDL: common bile duct ligation; DW: distilled water (control)

BW: body weight; MAP: mean arterial pressure; HR: heart rate; PP: portal pressure; CI: cardiac index: SVR: systemic vascular resistance; SMA: superior mesenteric artery.

In cirrhotic rats, curcumin significantly decreased PP, CI, and SMA flow. Curcumin also increased SVR and SMA resistance as compared with the DW-treated cirrhotic rats. Curcumin did not affect the hemodynamic parameters in sham-operated rats.

#### Plasma liver and kidney biochemistry parameters

The liver and kidney biochemistry parameters of experimental groups are shown in Table 2. There was no significant difference between curcumin and paralleled DW-treated groups.

**Table 2:** Plasma biochemistry parameters in sham or CBDL rats with DW or curcumin treatment

	Sham DW	Sham curcumin	CBDL DW	CBDL curcumin
**AST (U/l)**	109 ± 8	135 ± 11	662 ± 148^†^	822 ± 151^‡^
**ALT (U/l)**	58 ± 3	34 ± 4	140 ± 33^†^	192 ± 33^‡^
**Total bilirubin (mg/dl)**	<0.7*	<0.7*	6.03 ± 1.14^†^	8.20 ± 1.03^‡^
**BUN (mg/dl)**	24.3 ± 1.7	26.6 ± 1.6	21.4 ± 1.1	23.5 ± 3.5
**Creatinine (mg/dl)**	0.63 ± 0.11	0.53 ± 0.10	0.22 ± 0.03^†^	0.17 ± 0.00

Abbreviations: ALT, alanine transaminase; AST, aspartate transaminase; BUN, blood urea nitrogen.

* Under the detection limit;

† *P*<0.05, DW-treated CBDL group compared with DW-treated sham group;

‡ *P*<0.05, curcumin-treated CBDL group compared with curcumin-treated sham group.

AST: aspartate transaminase; ALT: alanine transaminase; BUN: blood urea nitrogen.

### Effects of curcumin on hepatic system

In the aforementioned experiment, curcumin reduced PP. The following experiments were therefore designed to evaluate if the portal hypotensive effect of curcumin was contributed via the hepatic system.

#### Liver fibrosis

**Figure 1:**
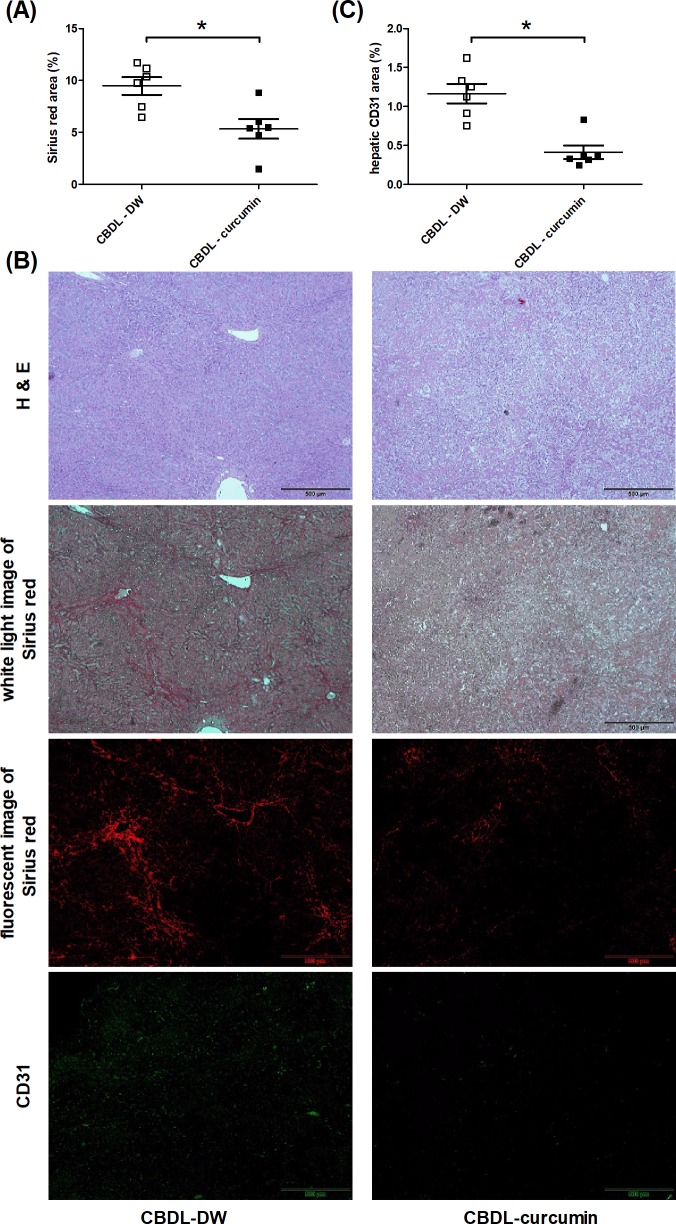
Curcumin reduced liver fibrosis and vascular density in rats with CBDL-induced cirrhosis (**A**) The extent of liver fibrosis evaluated by Sirius Red. The Sirius Red stained area, indicative of hepatic collagen fiber, was significantly lower in CBDL rats with curcumin treatment (CBDL-DW (vehicle, DW) compared with CBDL-curcumin *n*=6, 6). (**B**) The representative images of liver histological staining. The first panel: H&E staining images. As compared with CBDL-DW group, the curcumin-administered group showed less whitish fibrotic band. The second panel: Sirius Red staining images observed by white light, showing less Sirius Red stained area in curcumin-treated group. The third panel: Sirius Red stained images observed by fluorescence, showing less Sirius Red stained immunofluorescent area in curcumin-treated group. The fourth panel: CD31 (a vascular endothelial cell marker)-stained immunofluorescence images. (**C**) Hepatic vascular density determined by CD31 immunofluorescence staining was less in curcumin-treated group (*n*=6, 6). *P<0.05.

Figure 1A reveals that Sirius Red stained area was significantly less in CBDL rats with curcumin treatment as compared with the DW-treated CBDL rats. Figure 1B reveals the representative H&E and Sirius Red images of CBDL rats with curcumin or DW treatment. Liver cirrhosis was induced by CBDL, featured by the presence of bridging fibrosis and regeneration nodules. Curcumin significantly alleviated the severity of liver fibrosis. Sirius Red also revealed increased collagen in CBDL-DW rats with the characteristic deep-red (white light image) or orange-red color (immunofluoscent image), which was less in CBDL-curcurmin rats.

#### Hepatic vascular density

Figure 1C reveals that in CBDL rats treated with curcumin, the intrahepatic vascular area was significantly less than DW-treated CBDL rats. The representative image is shown in Figure 1B: the intrahepatic vascular area was represented by CD31 staining.

### Effects of curcumin on splanchnic system

In the aforementioned experiments, curcumin significantly alleviated splanchnic hemodynamic derangements. The following experiments were therefore designed to explore how curcumin affected splanchnic system.

### Mesenteric vascular density

**Figure 2:**
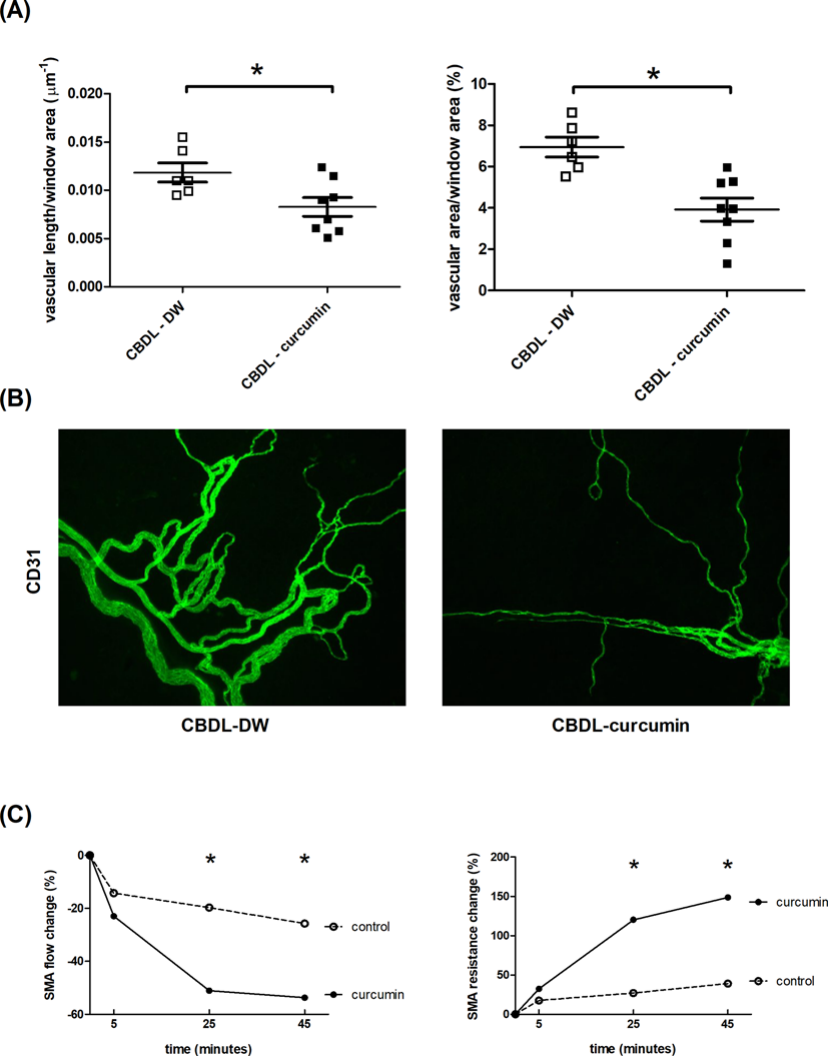
Curcumin reduced mesenteric angiogenesis and blood flow in rats with CBDL-induced liver cirrhosis (**A**) Mesenteric vascular density evaluated by CD31 (a vascular endothelial cell marker) immunofluorescence staining. Curcumin significantly reduced the mesenteric CD31-stained vascular area of the mesenteric window (*n*=6, 8). (**B**) The representative images of CD31-stained mesenteric windows. The stained vessels were less in curcumin-treated group. (**C**) The influence of curcumin on the flow and resistance of SMA (the representative vessel of splanchnic circulation) evaluated by Doppler flow probe. After 25 min of administration, curcumin significantly increased SMA resistance and decreased SMA flow (*n*=3, 3). *P<0.05.

Figure 2A shows that the vascular length and area per unit area of mesenteric window were significant lower in curcumin-treated CBDL rats as compared with the DW-treated CBDL rats. The representative CD31 staining images are shown in Figure 2B.

### Acute effects of curcumin on splanchnic hemodynamics

To further determine if curcumin affected splanchnic vascular contractility, the acute effects of curcumin on splanchnic hemodynamics were evaluated. In CBDL rats, a single dose of curcumin or DW was given. The changes in SMA flow and resistance were recorded at 5, 25, and 45 min after curcumin administration, respectively. Figure 2C reveals that curcumin decreased SMA flow and increased SMA resistance. The effects could be observed since 25 min after curcumin administration. This suggests that curcumin reduces splanchnic inflow by augmenting vascular contractility.

### Mesenteric protein expressions

**Figure 3:**
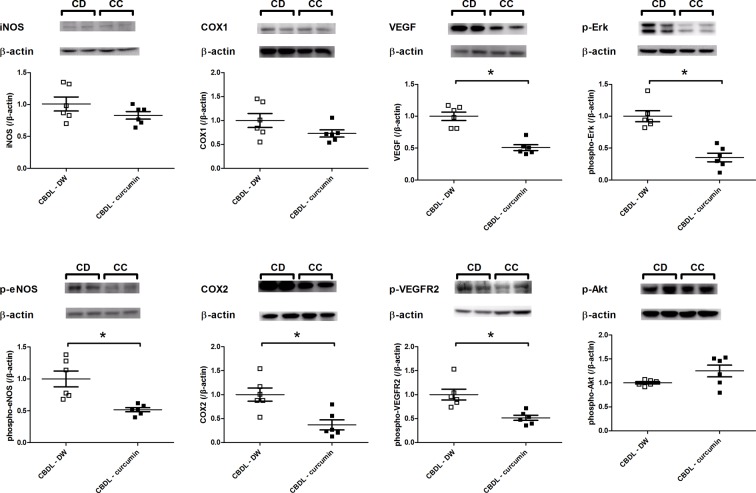
Curcumin significantly down-regulated the mesenteric proangiogenic and vasoactive factors protein expressions in rats with CBDL-induced liver cirrhosis The p-eNOS, COX2, VEGF, p-VEGFR2, and p-Akt protein expressions determined by Western blot were significantly down-regulated in CBDL rats with curcumin treatment (*n*=6, 6; CD, CBDL-DW; CC, CBDL-curcumin). *P<0.05.

Figure 3 discloses that p-endothelial nitric oxide synthase (eNOS), COX2, VEGF, p-VEGFR2, and p-Erk expressions significantly decreased in the mesentery of CBDL-curcumin rats. The protein expression of iNOS, COX1, and p-Akt was not influenced by curcumin.

### Effects of curcumin on collateral system

#### Portosystemic shunting

**Figure 4:**
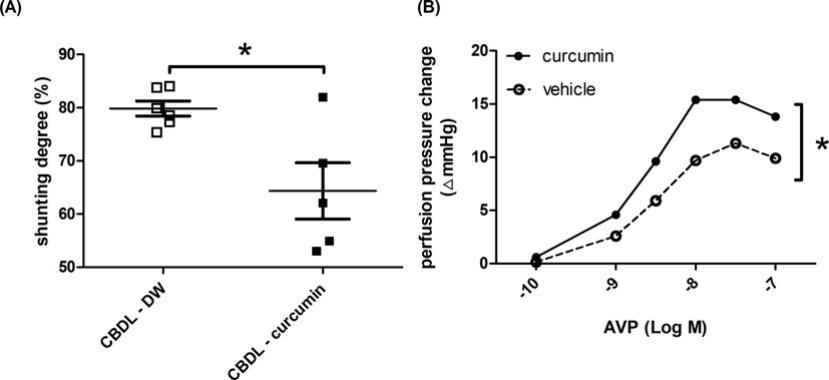
Curcumin reduced portosystemic collateral shunting degree and enhanced collateral vasoconstriction in rats with CBDL-induced liver cirrhosis (**A**) The shunting degree determined by color microsphere method was significantly lower in the curcumin-treated group (*n*=7, 5). (**B**) The portosystemic collateral vascular constriction evaluated by a collateral *in situ* perfusion model. As compared with vehicle (Krebs solution), curcumin preincubation significantly increased the perfusion pressure changes to AVP in the collateral vascular bed of CBDL rats (*n*=7, 5). *P<0.05.

Figure 4A depicts the severity of portosystemic shunting. Curcumin, as compared with DW, significantly attenuated the severity of shunting in CBDL rats (shunting degree: DW, curcumin (%): 79.8 ± 1.4, 64.3 ± 5.3).

#### Acute effect of curcumin on vascular contractility of collateral vascular bed

The effect of curcumin on collateral vascular bed was determined by *in situ* collateral perfusion (Figure 4B). Curcumin preincubation significantly increased the perfusion pressure changes to AVP in CBDL rats.

## Discussion

CBDL is a widely adopted cirrhotic animal model. Hepatic fibrosis could be observed since the second week after CBDL and liver cirrhosis established on the fourth week [21]. As a result, therapeutic agent given since the 14th day after CBDL is quite relevant to the clinical condition, when patients start to receive therapy after the detection of liver injury. In the present study, curcumin administered since the third to fourth week after CBDL effectively attenuated portal hypertension related abnormalities. Therefore, the use of curcumin is potentially beneficial in alleviating portal hypertension related complications. It is also worth noting that curcumin increased SVR and decreased CI, suggesting that curcumin corrected systemic hyperdynamic circulatory status in portal hypertension.

Portal hypertension is mainly determined by hepatic and splanchnic systems. Curcumin alleviated CCl_4_^−^ or alcohol-induced liver fibrosis in rats [22,23]. The antifibrotic effect was thought mainly through inhibition of hepatic stellate cell activation, the major triggering event in liver fibrosis [9]. Recent evidences also indicate that hepatic angiogenesis is linked to fibrogenesis [24], which can be ameliorated by curcumin: Yao et al. [25] had identified that curcumin ameliorated intrahepatic angiogenesis and sinusoid capillarization in cirrhotic rats. Zhang et al. [26] also reported that curcumin attenuated angiogenesis in liver fibrosis and inhibited angiogenic actions of hepatic stellate cells. In this study, curcumin was found to mitigate liver cirrhosis even being administered after fibrosis development. The antifibrotic effects of curcumin obviously contributed to improvement of portal hypertension.

In splanchnic system, both abnormal angiogenesis and vasodilatation drain excessive blood flow into portal system. Amongst the molecular mechanisms of pathological neovascularization in portal hypertension, VEGF is one of the most important factors [1]. Curcumin and turmeric attenuated arsenic-induced angiogenesis and inhibited VEGF expression in HCT-116 human colon cancer cells [27]. In mice fed with high-fat diet, curcumin supplementation reduced microvessel density in adipose tissue, which was coincided with VEGF and VEGFR2 down-regulation [28]. Decreased VEGF levels in conditioned media from cells treated with curcumin have also been noted [29]. VEGF induces angiogenesis via the activation of various signaling pathways such as phosphatidylinositol 3-kinase (PI3K)/Akt, protein kinase C (PKC), and mitogen-activated protein kinase (MAPK) cascades [30]. It has been found that curcumin inhibited Akt activation and down-regulated the expression of 5-lipooxygenase [31]. Prostaglandins synthesized by COX2 also participate in angiogenesis [32]. It has been reported that curcumin inhibited VEGF-induced *COX2* mRNA and protein expressions as well as PGE2 production in human intestinal microvascular endothelial cells [33]. Furthermore, NO plays a role in abdominal angiogenesis of portal hypertensive rats [34] and is the downstream molecule of VEGF signaling pathway [35]. Consistently, we identified that curcumin down-regulated mesenteric VEGF, p-VEGFR2, p-Erk, COX2, and p-eNOS protein expressions in cirrhotic rats, which are implicated in the alleviation of splanchnic angiogenesis and thus reduction in SMA flow and PP.

In cirrhosis, excess vasodilatory substances release, especially NO, results in peripheral and splanchnic vasodilatation, which is the functional component of splanchnic hyperemia. Effects of curcumin in NO synthases expression are controversial in different tissues under various pathophysiological conditions. Some studies have demonstrated that curcumin decreased intestinal NO level in rats with ischemia–reperfusion injury [36], and eNOS expression in endothelial cells [37]. Curcumin also improved survival in rats with thioacetamide-induced hepatotoxicity by inhibiting the nuclear binding of NF-kB and iNOS protein expression [38]. In this study, acute curcumin administration was found to decrease SMA flow and increase SMA resistance. Chronic curcumin treatment also down-regulated mesenteric eNOS activation. This suggested that curcumin reduces splanchnic flow, not only by inhibiting angiogenesis, but also through restoring the vascular contractility via the inhibition of mesenteric eNOS phosphorylation.

The portosystemic collateral vessels develop with the aim to divert the stagnant splanchnic and portal blood flow. However, it brings lethal complications such as gastroesophagel variceal hemorrhage. In the present study, curcumin administration significantly attenuated shunting degree. This may be due to curcumin-induced reduction in hepatic fibrosis and splanchnic inflow. Furthermore, curcumin was also demonstrated to induce collateral vasoconstriction. The data suggest that curcumin are potentially beneficial in the control of shunting-related complications.

There is a limitation of the present study. Chronic curcumin administration in cirrhotic rats alleviates portal hypertension related hemodynamic derangements and portosystemic collaterals. As curcumin ameliorated liver cirrhosis, mitigated portal hypertension and splanchnic vascular bed dilatation are highly expected. However, only acute experiment was applied to test the effect of curcumin on splanchnic vasculature in the current study.

**Figure 5:**
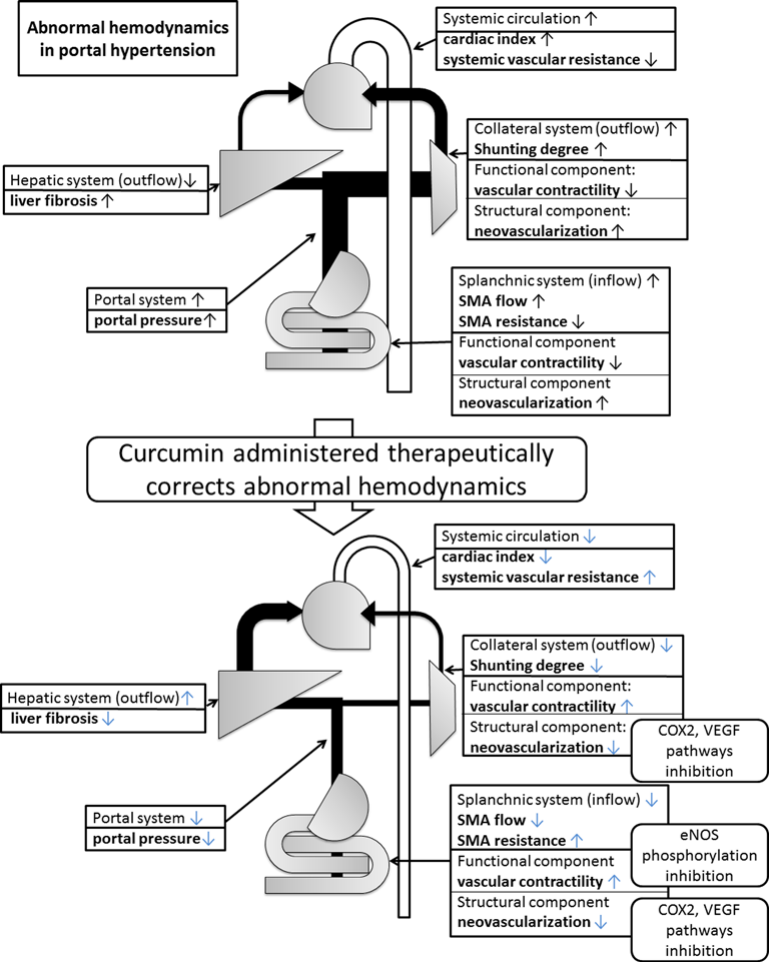
Beneficial effects of curcumin on liver cirrhosis and portal hypertension In liver cirrhosis, portal hypertension develops due to the abnormal hemodynamic changes in liver, splanchnic systems, and collaterals. Curcumin attenuates portal hypertension by alleviating the hepatic fibrosis, extrahepatic angiogenesis, and hyperdynamic circulation. The beneficial effects over splanchnic and collateral vascular beds are at least partly through the inhibition of VEGF, COX2, and eNOS pathways.

In conclusion, chronic curcumin treatment alleviated portal hypertension (Figure 5). This was due to ameliorated hepatic fibrosis and splanchnic inflow. The beneficial effects of curcumin on splanchnic inflow were due to the attenuation of abnormal angiogenesis and restoring vascular contractility, which were through inhibition of VEGF signaling, in which COX and NOS were also implicated. Due to the aforementioned effects, portosystemic collaterals were also alleviated. Curcumin, a traditional seasoning without significant toxicity, even though administered after liver fibrosis has been developed, exerts potential benefits in the treatment of liver cirrhosis and portal hypertension.

## Clinical perspectives

- Portal hypertension induced by liver cirrhosis results in lethal complication. Angiogenesis and vasodilatation are important pathological factors, which may be affected by curcumin.
- Curcumin attenuated portal hypertension in cirrhotic rats via inducing vasoconstriction in splanchnic system and decreasing mesenteric angiogenesis through inhibition of eNOS and VEGF pathway, respectively.
- Clinical investigations based on the present study may contribute to a better control of portal hypertension in cirrhotic patients.

## Abbreviations

AVP: arginine vasopressin
BW: body weight
CBDL: common bile duct ligation
CI: cardiac index
CO: cardiac output
COX: cyclooxygenase
DW: distilled water
eNOS: endothelial nitric oxide synthase
H&E: Hematoxylin and Eosin
iNOS: inducible nitric oxide synthase
MAP: mean arterial pressure
PP: portal pressure
SMA: superior mesenteric artery
SMV: superior mesenteric vein
SVR: systemic vascular resistance
VEGF: vascular endothelial growth factor
VEGFR2: VEGF receptor 2

## References

[1] Fernandez M., Vizzutti F., Garcia-Pagan J.C., Rodes J. and Bosch J. (2004) Anti-VEGF receptor-2 monoclonal antibody prevents portal-systemic collateral vessel formation in portal hypertensive mice. Gastroenterology 126, 886–894 14988842 10.1053/j.gastro.2003.12.012

[2] Mejias M., Garcia-Pras E., Tiani C., Miquel R., Bosch J. and Fernandez M. (2009) Beneficial effects of sorafenib on splanchnic, intrahepatic, and portocollateral circulations in portal hypertensive and cirrhotic rats. Hepatology 49, 1245–1256 19137587 10.1002/hep.22758

[3] Huang H.C., Wang S.S., Hsin I.F., Chang C.C., Lee F.Y., Lin H.C. (2012) Cannabinoid receptor 2 agonist ameliorates mesenteric angiogenesis and portosystemic collaterals in cirrhotic rats. Hepatology 56, 248–258 22290687 10.1002/hep.25625

[4] Hsu S.J., Lee F.Y., Wang S.S., Hsin I.F., Lin T.Y., Huang H.C. (2015) Caffeine ameliorates hemodynamic derangements and portosystemic collaterals in cirrhotic rats. Hepatology 61, 1672–1684 25557829 10.1002/hep.27679

[5] Solà E., Lens S., Guevara M., Martín-Llahí M., Fagundes C., Pereira G. (2010) Hyponatremia in patients treated with terlipressin for severe gastrointestinal bleeding due to portal hypertension. Hepatology 52, 1783–1790 20931555 10.1002/hep.23893

[6] Huang H.C., Chu C.J., Lee F.Y., Chang F.Y., Wang S.S., Lin H.C. (2000) Chronic inhibition of nitric oxide ameliorates splanchnic hyposensitivity to glypressin in a hemorrhage-transfused rat model of portal hypertension. Scand. J. Gastroenterol. 35, 1308–1313 11199372 10.1080/003655200453674

[7] Chen Y.R. and Tan T.H. (1998) Inhibition of the c-Jun N-terminal kinase (JNK) signaling pathway by curcumin. Oncogene 17, 173–178 9674701 10.1038/sj.onc.1201941

[8] Ammon H.P. and Wahl M.A. (1991) Pharmacology of *Curcuma longa*. Planta Med. 57, 1–7 2062949 10.1055/s-2006-960004

[9] Vera-Ramirez L., Pérez-Lopez P., Varela-Lopez A., Ramirez-Tortosa M., Battino M. and Quiles J.L. (2013) Curcumin and liver disease. Biofactors 39, 88–100 23303639 10.1002/biof.1057

[10] Arbiser J.L., Klauber N., Rohan R., van Leeuwen R., Huang M.T., Fisher C. (1998) Curcumin is an *in vivo* inhibitor of angiogenesis. Mol. Med. 4, 376–383 10780880 PMC2230271

[11] Farhangkhoee H., Khan Z.A., Chen S. and Chakrabarti S. (2006) Differential effects of curcumin on vasoactive factors in the diabetic rat heart. Nutr. Metab. (Lond). 3, 27 16848894 10.1186/1743-7075-3-27 PMC1543622

[12] Franco F., Gigou M., Szekely A.M. and Bismuth H. (1979) Portal hypertension after bile duct obstruction: effect of bile diversion on portal pressure in the rat. Arch. Surg. 114, 1064–1067 485838 10.1001/archsurg.1979.01370330086016

[13] Cameron G.R. and Muzaffar H.S. (1958) Disturbances of structure and function in the liver as the result of biliary obstruction. J. Pathol. Bacteriol. 75, 333–349 13576315 10.1002/path.1700750212

[14] Lee F.Y., Colombato L.A., Albillos A. and Groszmann R.J. (1993) Administration of Nω-nitro-L- arginine ameliorates portal-systemic shunting in portal-hypertensive rats. Gastroenterology 105, 1464–1470 8224649 10.1016/0016-5085(93)90152-3

[15] Lee F.Y., Wang S.S., Tsai Y.T., Lin H.J., Lin H.C., Chu C.J. (1997) Aminoguanidine corrects hyperdynamic circulation without ameliorating portal hypertension and portal hypertensive gastropathy in anesthetized portal hypertensive rats. J. Hepatol. 26, 687–693 9075678 10.1016/s0168-8278(97)80436-9

[16] Albillos A., Colombato L.A. and Groszmann R.J. (1992) Vasodilatation and sodium retention in prehepatic portal hypertension. Gastroenterology 102, 931–935 1537529 10.1016/0016-5085(92)90179-3

[17] Bradford M.M. (1976) A rapid and sensitive method for the quantitation of microgram quantities of protein utilizing the principle of protein-dye binding. Anal. Biochem. 72, 248–254 942051 10.1016/0003-2697(76)90527-3

[18] Chojkier M. and Groszmann R.J. (1981) Measurement of portal-systemic shunting in the rat by using γ-labeled microspheres. Am. J. Physiol. 240, G371–G375 7235023 10.1152/ajpgi.1981.240.5.G371

[19] Hodeige D., de Pauw M., Eechaute W., Weyne J. and Heyndrickx G.R. (1999) On the validity of blood flow measurement using colored microspheres. Am. J. Physiol. 276, H1150–H1158 10199837 10.1152/ajpheart.1999.276.4.H1150

[20] Chan C.C., Lee F.Y., Wang S.S., Chang F.Y., Lin H.C., Chu C.J. (1999) Effects of vasopressin on portal-systemic collaterals in portal hypertensive rats: role of nitric oxide and prostaglandin. Hepatology 30, 630–635 10462367 10.1002/hep.510300317

[21] Kountouras J., Billing B.H. and Scheuer P.J. (1984) Prolonged bile duct obstruction: a new experimental model for cirrhosis in the rat. Br. J. Exp. Pathol. 65, 305–311 6743531 PMC2040968

[22] Zhao Y., Ma X., Wang J., He X., Hu Y., Zhang P. (2014) Curcumin protects against CCl4-induced liver fibrosis in rats by inhibiting HIF-1α through an ERK-dependent pathway. Molecules 19, 18767–18780 25407718 10.3390/molecules191118767 PMC6270950

[23] Chen N., Geng Q., Zheng J., He S., Huo X. and Sun X. (2014) Suppression of the TGF-β/Smad signaling pathway and inhibition of hepatic stellate cell proliferation play a role in the hepatoprotective effects of curcumin against alcohol-induced hepatic fibrosis. Int. J. Mol. Med. 34, 1110–1116 25069637 10.3892/ijmm.2014.1867

[24] Iwakiri Y., Shah V. and Rockey D.C. (2014) Vascular pathobiology in chronic liver disease and cirrhosis - current status and future directions. J. Hepatol. 61, 912–924 24911462 10.1016/j.jhep.2014.05.047 PMC4346093

[25] Yao Q., Lin Y., Li X., Shen X., Wang J. and Tu C. (2013) Curcumin ameliorates intrahepatic angiogenesis and capillarization of the sinusoids in carbon tetrachloride-induced rat liver fibrosis. Toxicol. Lett. 222, 72–82 23845850 10.1016/j.toxlet.2013.06.240

[26] Zhang F., Zhang Z., Chen L., Kong D., Zhang X., Lu C. (2014) Curcumin attenuates angiogenesis in liver fibrosis and inhibits angiogenic properties of hepatic stellate cells. J. Cell. Mol. Med. 18, 1392–1406 24779927 10.1111/jcmm.12286 PMC4124023

[27] Pantazis P., Varman A., Simpson-Durand C., Thorpe J., Ramalingam S., Subramaniam D. (2010) Curcumin and turmeric attenuate arsenic-induced angiogenesis *in ovo*. Altern. Ther. Health Med. 16, 12–14 20232614

[28] Ejaz A., Wu D., Kwan P. and Meydani M. (2009) Curcumin inhibits adipogenesis in 3T3-L1 adipocytes and angiogenesis and obesity in C57/BL mice. J. Nutr. 139, 919–925 19297423 10.3945/jn.108.100966

[29] Gururaj A.E., Belakavadi M., Venkatesh D.A., Marmé D. and Salimath B.P. (2002) Molecular mechanisms of anti-angiogenic effect of curcumin. Biochem. Biophys. Res. Commun. 297, 934–942 12359244 10.1016/s0006-291x(02)02306-9

[30] Zachary I. (2003) VEGF signalling: integration and multi-tasking in endothelial cell biology. Biochem. Soc. Trans. 31, 1171–1177 14641020 10.1042/bst0311171

[31] Aggarwal S., Ichikawa H., Takada Y., Sandur S.K., Shishodia S. and Aggarwal B.B. (2006) Curcumin (diferuloylmethane) down-regulates expression of cell proliferation and antiapoptotic and metastatic gene products through suppression of IkappaBalpha kinase and Akt activation. Mol. Pharmacol. 69, 195–206 16219905 10.1124/mol.105.017400

[32] Klenke F.M., Gebhard M.M., Ewerbeck V., Abdollahi A., Huber P.E. and Sckell A. (2006) The selective Cox-2 inhibitor celecoxib suppresses angiogenesis and growth of secondary bone tumors: an intravital microscopy study in mice. BMC. Cancer 6, 9 16409625 10.1186/1471-2407-6-9 PMC1360103

[33] Binion D.G., Otterson M.F. and Rafiee P. (2008) Curcumin inhibits VEGF-mediated angiogenesis in human intestinal microvascular endothelial cells through COX-2 and MAPK inhibition. Gut 57, 1509–1517 18596194 10.1136/gut.2008.152496 PMC2582343

[34] Sumanovski L.T., Battegay E., Stumm M., van der Kooij M. and Sieber C.C. (1999) Increased angiogenesis in portal hypertensive rats: role of nitric oxide. Hepatology 29, 1044–1049 10094944 10.1002/hep.510290436

[35] Zachary I. and Gliki G. (2001) Signaling transduction mechanisms mediating biological actions of the vascular endothelial growth factor family. Cardiovasc. Res. 49, 568–581 11166270 10.1016/s0008-6363(00)00268-6

[36] Karatepe O., Gulcicek O.B., Ugurlucan M., Adas G., Battal M., Kemik A. (2009) Curcumin nutrition for the prevention of mesenteric ischemia-reperfusion injury: an experimental rodent model. Transplant. Proc. 41, 3611–3616 19917353 10.1016/j.transproceed.2009.08.002

[37] Rossig L., Li H., Fisslthaler B., Urbich C., Fleming I., Förstermann U. (2002) Inhibitors of histone deacetylation downregulate the expression of endothelial nitric oxide synthase and compromise endothelial cell function in vasorelaxation and angiogenesis. Circ. Res. 91, 837–844 12411399 10.1161/01.res.0000037983.07158.b1

[38] Shapiro H., Ashkenazi M., Weizman N., Shahmurov M., Aeed H. and Bruck R. (2006) Curcumin ameliorates acute thioacetamide-induced hepatotoxicity. J. Gastroenterol. Hepatol. 21, 358–366 16509859 10.1111/j.1440-1746.2005.03984.x

